# Remdesivir in the Treatment of COVID-19: A Propensity Score-Matched Analysis from a Public Hospital in New York City Assessing Renal and Hepatic Safety

**DOI:** 10.3390/jcm11113132

**Published:** 2022-05-31

**Authors:** Hyomin Lim, Leonidas Palaiodimos, Cesar G. Berto, Oluwatitomi Tedunjaiye, Paras Malik, Sanjana Nagraj, Hansol Choi, Nang San Hti Lar Seng, Michail Kladas, Amrin Kharawala, Dimitrios Karamanis, Nidhi Varma, Acharya Anjali

**Affiliations:** 1 Albert Einstein College of Medicine, Bronx, New York, NY 10461, USA; limh6@nychhc.org (H.L.); bertomc@nychhc.org (C.G.B.); tedunjao@nychhc.org (O.T.); paras.malik@nychhc.org (P.M.); nagrajs@nychhc.org (S.N.); hansolc@nychhc.org (H.C.); htilarn@nychhc.org (N.S.H.L.S.); kladasm@nychhc.org (M.K.); kharawaa@nychhc.org (A.K.); varman@nychhc.org (N.V.); anjali.acharya@nychhc.org (A.A.); 2 Department of Medicine, Jacobi Medical Center, NYC Health + Hospitals, New York, NY 10461, USA; dkaramanis@hotmail.com; 3 Division of Hospital Medicine, Jacobi Medical Center, NYC Health + Hospitals, New York, NY 10461, USA; 4 CUNY School of Medicine, New York, NY 10031, USA; 5 Department of Medicine, North Central Bronx Hospital, NYC Health + Hospitals, New York, NY 10467, USA; 6 Department of Health Informatics, Rutgers School of Health Professions, Newark, NJ 07102, USA; 7 Department of Economics, University of Piraeus, 18534 Piraeus, Greece; 8 Division of Nephrology, Jacobi Medical Center, NYC Health + Hospitals, New York, NY 10461, USA

**Keywords:** remdesivir, COVID-19, safety, adverse events, acute kidney injury, nephrotoxicity

## Abstract

While the relative efficacy of remdesivir as a therapeutic agent in selected patients with COVID-19 has been established, safety concerns have been raised regarding potential nephrotoxicity and hepatotoxicity. Our main objective was to investigate the kidney- and liver-related safety outcomes in patients with COVID-19 treated with remdesivir in a public hospital in New York. A propensity score-matched retrospective study was conducted in hospitalized patients with COVID-19 from 1 June 2020 to 10 March 2021. A total of 927 patients were included in this study (remdesivir: 427, non-remdesivir: 500; women: 51.8%; median age 61 years; median BMI: 28.5 kg/m^2^). Matching without replacement yielded a cohort of 248 patients (124 in each group). In the matched cohort, the remdesivir group had a significantly lower rate of acute kidney injury (AKI) (12.1% vs. 21.8%, *p* = 0.042), a lower rate of acute liver injury (ALI) on the verge of statistical significance (7.3% vs. 14.5%, *p* = 0.067), and non-significantly lower death rate (13.7% vs. 16.1%, *p* = 0.593) compared to the non-remdesivir group. Multivariable analyses revealed that patients treated with remdesivir were found to be associated with a significantly lower likelihood for AKI (OR: 0.40; 95% CI: 0.24–0.67, *p* < 0.001), no association was found for ALI (OR: 0.68; 95% CI: 0.35–1.30, *p* = 0.241), while a trend towards an association of patients treated with remdesivir with a lower likelihood for in-hospital death was observed (OR: 0.57; 95% CI: 0.32–1.01, *p* = 0.053). In conclusion, no safety concerns with regards to renal and liver outcomes were raised in patients with COVID-19 treated with remdesivir. Instead, there were signals of possible nephroprotection and improved in-hospital mortality.

## 1. Introduction

Viral replication is the main characteristic of the early infection with the severe acute respiratory syndrome coronavirus 2 (SARS-CoV-2) and additionally plays a central role in the subsequent pulmonary phase of Coronavirus Disease 2019 (COVID-19) [1]. Therefore, remdesivir, which had originally been developed for the treatment of Ebola virus disease and found to inhibit the replication of various coronaviruses in preclinical studies, was one of the first therapeutics to receive attention at the beginning of the pandemic [2].

Remdesivir is an inhibitor of the RNA-dependent RNA polymerase, which is essential for viral replication [3]. It is phosphorylated by cellular kinases to form the pharmacologically active nucleoside triphosphate that can be integrated into viral RNA-dependent polymerase, which then induces premature termination of viral RNA transcription [3]. In the ACTT-1 trial, hospitalized patients with COVID-19 that received remdesivir had a significantly shorter recovery time, higher likelihood of clinical improvement at day 15, and non-significant lower death rate by day 29 compared to patients that received placebo [4]. The preliminary findings of ACTT-1 findings made remdesivir the first drug to receive emergency use authorization by the FDA initially for the treatment of patients with severe COVID-19 [5]. In contrast, the WHO Solidarity trial did not show positive results [6]. However, a subsequent meta-analysis for the American College of Physicians revealed that remdesivir offered mortality benefits in patients that were on supplemental oxygen but not on mechanical ventilation [7]. Finally, the recently published PINETREE trial showed that an early three-day course of remdesivir decreased substantially the risk of hospitalization in outpatients with risk factors for COVID-19 progression and led to an expansion of indications for use of remdesivir [8,9].

While the relative efficacy of remdesivir as a therapeutic agent in selected patients with COVID-19 has been established, safety concerns have been raised mainly regarding potential nephrotoxicity and hepatotoxicity [10,11,12]. The possible mechanism behind the presumed nephrotoxicity of remdesivir is the prolonged plasma half-life of its metabolites and the accumulation of sulfobutylether-*β*-cyclodextrin (SBECD) carrier which is the solubilizing excipient used to prepare the intravenous formulation as remdesivir has limited water solubility [13]. While remdesivir itself may not be nephrotoxic, there are concerns that SBECD accumulation in tubular cells may be responsible for renal injury [4,13]. In the ACTT-1 trial, the Acute Kidney Injury (AKI) rates were similar in the remdesivir and placebo groups, 3.9% and 4.1%, respectively [4]. In the PINETREE trial, the mean change from baseline in creatinine clearance was lower in the remdesivir group compared to the placebo group (0.26 ± 21.2 mL per minute vs 1.9 ± 18.6 mL per minute) [8]. However, both landmark randomized studies excluded patients with creatinine clearance <30 mL/min, Refs. [4,8] while available real-world studies are small or obtained data from adverse events reporting system databases [14,15,16].

Potential remdesivir-induced liver injury has been another safety concern [17,18]. The metabolism of remdesivir occurs via CYP3A4 in the liver, which can be one of the targeted organs by SARS-CoV-2 since Angiotensin-converting enzyme 2 (ACE2) is present in hepatocytes and cholangiocytes [19]. No liver-related safety signals were detected in ACTT-1 and PINETREE trials but patients with significant elevation of liver enzymes at baseline were excluded [4,8].

Therefore, well-designed real-world studies are needed to further assess the renal and liver outcomes of patients on remdesivir with special emphasis on patients with AKI, chronic kidney, or liver disease. The primary objective of this analysis was to investigate the kidney- and liver-related safety outcomes of patients with COVID-19 treated with remdesivir in a public hospital in the Bronx, New York. Our secondary objective was to investigate the efficacy of remdesivir with regard to hard in-hospital outcomes.

## 2. Materials and Methods

### 2.1. Study Design, Study Setting, Patient Population

This was a propensity score-matched observational cohort study performed at New York City Health and Hospitals/Jacobi, an inner-city hospital in the Bronx, New York. Patients ≥ 18 years of age who were admitted to an inpatient service, including the intensive care unit (ICU), with laboratory-confirmed COVID-19 from 1 June 2020 to 10 March 2021 were included. We excluded patients who met any one of the following criteria: (i) patients < 18 years old; (ii) patients without laboratory-confirmed COVID-19; (iii) patients who were still hospitalized at the time of data collection; (iv) women who were pregnant at the time of the index hospitalization. Per our institutional protocol for the diagnosis and management of COVID-19, all patients had to be tested for COVID-19 immediately upon arrival to the emergency room and no remdesivir could be initiated without approval, for which laboratory confirmation of COVID-19 was needed. The study was approved by the Biomedical Research Alliance of New York (BRANY) Institutional Review Board with a waiver of informed consent (IRB #20-12-103-373). Data were fully de-identified and anonymized before the data was accessed and the IRB waived the requirement for informed consent.

### 2.2. Data Sources

Study data were obtained from electronic health records via appropriate diagnostic codes (Epic Systems, Verona, WI, USA). The initial dataset was reviewed by two independent investigators for accuracy (HL and SN). Two pairs of additional independent investigators reviewed individual charts to obtain additional information (LP-CB, MP-NV). The extracted data included age, gender, body mass index (BMI), history of hypertension, hyperlipidemia, diabetes, coronary artery disease (CAD), congestive heart failure (CHF), stroke, chronic kidney disease (CKD) including stage, end-stage renal disease (ESRD), chronic liver disease (none, alcohol hepatitis, hepatitis B or C), liver cirrhosis (none, compensated, decompensated), sequential laboratory data including blood urea nitrogen (BUN), creatinine (Cr), albumin, total bilirubin, alkaline phosphatase (ALP), aspartate transaminase (AST) and alanine transaminase (ALT), COVID-19 severity on presentation, remdesivir administration during the index hospitalization (our institutional guidelines suggested a treatment duration of up to five days with the option to extend to up to ten days for patients with critical COVID-19), and outcomes including invasive mechanical ventilation, admission to intensive care unit (ICU), acute kidney injury (AKI), initiation of dialysis, or acute liver injury (ALI) during the index hospitalization, death, and hospital discharge. COVID-19 severity on presentation was adjudicated by two independent attending physicians (LP and AA) based on the NIH COVID-19 treatment guidelines (moderate: evidence of lower respiratory disease and oxygen saturation ≥94% on room air; severe: oxygen saturation <94% on room air; critical: respiratory failure requiring intubation and/or multiple organ dysfunction requiring ICU admission) [20]. AKI during the index hospitalization was adjudicated by two independent nephrologists (NV and AA) based on Kidney Disease: Improving Global Outcomes (KDIGO) guidelines on AKI (AKI of any stage was defined by an increase in serum creatinine by 0.3 mg/dL or more within 48 hours or an increase in serum creatinine to 1.5 times baseline or more within the last 7 days; stage 2 AKI was defined by an increase in serum creatinine 2–2.9 times baseline, and stage 3 AKI by an increase in serum creatinine more than three times baseline or increase to ≥ 4 mg/dL or need for initiation of renal replacement therapy; accurate data on urine output were not expected to be available) [21]. The glomerular filtration rate was estimated based on the CKD-EPI equation. ALI during the index hospitalization was adjudicated by two independent attending physicians (LP and NP) based on serum ALT and/or AST levels equal to or greater than 2.5 times the upper limit normal level which corresponds to grade 2 moderate liver injury [22]. Baseline laboratory tests were obtained while patients were located in the emergency room and before initiation of any medication as defined by our institutional protocol regarding COVID-19 treatment. The data were processed and analyzed without any personal identifiers to maintain patient confidentiality as per the Health Insurance Portability and Accountability Act (HIPAA).

### 2.3. Exposure of Interest and Outcomes

The exposure of interest was remdesivir. Patients were classified into two groups based on remdesivir administration: patients that received remdesivir and patients that did not receive remdesivir. The primary endpoints were AKI and ALI. The secondary endpoints were initiation of dialysis, invasive mechanical ventilation, admission to ICU, and in-hospital death.

### 2.4. Statistical Analysis

Propensity score matching was conducted to create comparable groups [23]. The propensity scores were estimated using a logistic regression model, in which fourteen covariates were used: age, gender, BMI, hypertension, hyperlipidemia, diabetes, CAD, CHF, stroke, CKD, chronic liver disease, liver cirrhosis, chronic alcohol use disorder, and COVID-19 severity on admission. The estimated propensity score was the predicted probability of receiving remdesivir derived from the fitted model.

We performed a nearest-neighbor matching without (one-to-one) replacement. Once a remdesivir-treated patient had been matched with a non-remdesivir patient, the latter was no longer available as a potential match for subsequent remdesivir patients. An optimal caliper width of 0.2 of the pooled standard deviation of the logit of the propensity score was used [24].

Continuous data were presented as median with interquartile range (IQR) and categorical data as absolute and relative frequencies. The *t*-test was used to compare continuous variables and chi-square for dichotomous variables. To further assess the balance of covariates between the remdesivir and non-remdesivir groups before and after propensity-score matching, standardized mean differences (SMD) were also calculated. In contrast to significance testing, SMD does not depend upon the size of the sample [25]. A standardized mean difference lower than the absolute value of 10% was considered to support the assumption of balance between groups.

For both cohorts (before matching and after matching without replacement) the outcomes of mortality, AKI, and ALI were compared between groups using logistic regression models resulting in an odds ratio (OR) with a 95% confidence interval. We applied univariate analyses and one multivariate model for each cohort and outcome that included remdesivir and baseline characteristics that were found significant (*p* < 0.05) in the univariate.

Statistical analysis was performed with STATA (version 14·1; STATA Corporation, College Station, TX, USA) and for matching, the psmatch2 module was used [26]. A nominal *p*-value < 0.05 was considered statistically significant.

## 3. Results

### 3.1. Baseline Patient Characteristics

**Figure 1:**
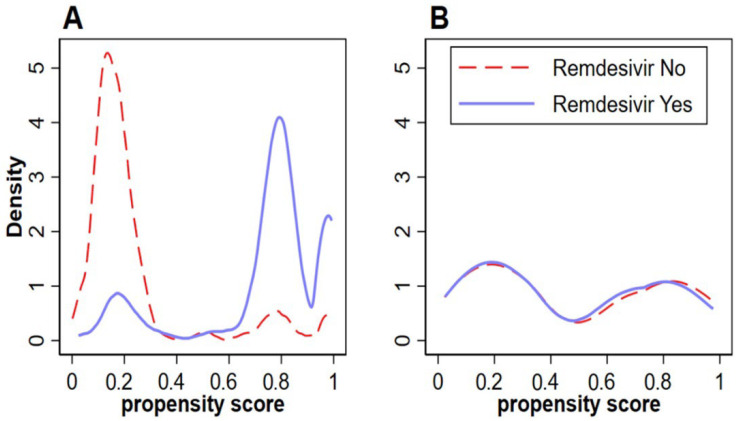
Density of Propensity Scores (**A**): Before Matching, (**B**): After Matching without Replacement.

In total, 927 patients were included in this study (remdesivir: 427, non-remdesivir: 500), 480 women (51.8%) and 447 men (48.2%). The median age was 61 (IQR 47–73) years and the median BMI was 28.5 (IQR 24.4–33.5) kg/m^2^. Hypertension, diabetes, and hyperlipidemia were the most common comorbidities being prevalent in 56.9%, 38.9%, and 26.5% of our patients, respectively. A total of 12.5% had CKD IIIA-V or ESRD on dialysis (CKD IIIA: 5.7%, CKD IIIB: 2.7% had CKD IV: 1.2%, CKD V: 1.9%, ESRD on dialysis: 1%). A total of 3% had chronic liver disease and 1.6% had liver cirrhosis. Regarding COVID-19 severity on admission, 52.5% were considered to have moderate disease, 35% had severe disease, and 11.8% had critical COVID-19. The rate of severe or critical COVID-19 in the remdesivir group was significantly higher compared to the non-remdesivir group (*p* < 0.001). Matching without replacement yielded a cohort of 248 patients. There were no missing data. Detailed baseline patient characteristics of the original cohort and the cohort after matching without replacement are presented in Table 1 and the density of propensity scores is presented in Figure 1.

### 3.2. Laboratory Markers on Presentation

In the overall cohort, median BUN was 15 (IQR 11–24) mg/dL, median Cr was 1.0 (IQR 0.8–1.3) mg/dL, median AST was 34 (IQR 24–60) U/L, and median ALT was 26 (IQR 16–45.5) U/L. Concentrations of baseline laboratory markers of the original cohort and the cohort after matching without replacement are presented in Table 2.

### 3.3. Outcomes

In-hospital, AKI was observed in 13.2% of patients in the original cohort (remdesivir 15.5%, non-remdesivir 11.2%, *p* = 0.055). After matching, AKI had a significantly lower rate in the remdesivir group compared to the non-remdesivir group (12.1% vs. 21.8%, *p* = 0.042). Only 9 patients (0.9%) required initiation of dialysis without significant differences observed in the original cohort or the cohort after matching. The mean serum creatinine was decreased from 1.37 mg/dL before treatment with remdesivir to 1.21 mg/dL after completion of treatment with remdesivir in the cohort before matching (*p* < 0.001) and from 1.34 mg/dL to 1.19 mg/dL in the cohort after matching (*p* = 0.007). ALI was observed in 8.2% of patients in the original cohort (remdesivir 9.6%, non-remdesivir 7%, *p* = 0.150). After matching, a signal towards lower ALI incidence in the remdesivir group compared to the non-remdesivir group was noted (7.3% vs. 14.5%, *p* = 0.067). In the overall cohort, a total of 11.9% died during hospitalization, 12.7% required intubation, and 19.3% required admission to the ICU. The rates of in-hospital death, intubation, and ICU admission were significantly higher in the remdesivir group (18%, 19.4%, 28.8%, respectively) compared to the non-remdesivir group (6.6%, 7%, 11.2%, respectively) (*p* < 0.001). After matching, however, the rates of in-hospital death, intubation, and ICU admission were higher in the non-remdesivir group (16.1%, 19.4%, 25%, respectively) compared to the remdesivir group (13.7%, 11.3%, 21.8%, respectively) but these differences were not statistically significant. In-hospital outcomes and AKI per stage are presented in Table 3 and Supplementary Table S1 respectively.

Subgroup analyses of the patients with CKD and chronic liver disease for the outcomes of AKI and ALI, respectively, were conducted. In the matched cohort, the AKI rates were similar in patients that received remdesivir to those that did not receive it (27.3% vs. 26.7%, *p* = 0.967). One patient developed ALI (1/4) among patients with chronic liver disease that did not receive remdesivir in the cohort after matching and no patients among those that were treated with remdesivir (0/4). The subgroup analysis is presented in Table 4.

### 3.4. Logistic Regression Analyses

#### 3.4.1. Acute Kidney Injury

In the multivariable analysis for the outcome of AKI, patients treated with remdesivir were found to be associated with a significantly lower likelihood of AKI (OR: 0.40; 95% CI: 0.24–0.67, *p* < 0.001) in the overall cohort, while the association was on the verge of statistical significance in the smaller post-matching cohort (OR: 0.48; 95% CI: 0.23–1.01, *p* = 0.054). Higher age group, hypertension, and higher COVID-19 severity on presentation were all associated with a higher likelihood of AKI. The univariate and multivariate analyses for the outcome of AKI in the overall and post-matching cohorts are presented in Table 5.

#### 3.4.2. Acute Liver Injury

In the multivariable analysis for the outcome of ALI, no association between treatment with remdesivir and ALI was noted in either cohort (overall cohort OR: 0.68; 95% CI: 0.35–1.30, *p* = 0.241; post-matching cohort OR: 0.47; 95% CI: 0.20–1.11, *p* = 0.087). Higher COVID-19 severity on presentation was the only variable associated with a higher likelihood for ALI in this analysis, whereas female sex and hypertension were the only variables associated with a lower likelihood for ALI. The univariate and multivariate analyses for the outcome of ALI in the overall and post-matching cohorts are presented in Table 6.

#### 3.4.3. Mortality

In the multivariable analysis for the outcome of in-hospital mortality, a trend towards an association of patients treated with remdesivir with a lower likelihood for in-hospital death was observed in the overall cohort (OR: 0.57; 95% CI: 0.32–1.01, *p* = 0.053) that was lost in the smaller post-matching cohort (OR: 0.97; 95% CI: 0.42–2.22, *p* = 0.941). Higher age group, CKD/ESRD, and higher COVID-19 severity on presentation were associated with a higher likelihood of death. The univariate and multivariate analyses for the outcome of death in the overall and post-matching cohorts are presented in Table 7.

## 4. Discussion

Our propensity score-matched study investigated the renal and liver safety outcomes and in-hospital mortality of patients treated with remdesivir in a cohort of 927 patients admitted with COVID-19 in a public hospital in the Bronx, New York. We found that the remdesivir group had a significantly lower rate of AKI and remdesivir was associated with a lower likelihood for AKI. In addition, an indication towards lower ALI rates in the remdesivir group was observed, while remdesivir itself was not associated with a higher or lower likelihood of ALI. Patients with CKD and chronic liver disease that were treated with remdesivir did not have higher rates of AKI or ALI, respectively, compared to those that did not receive remdesivir. Regarding in-hospital mortality, the remdesivir group had a non-significantly lower death rate compared to the non-remdesivir group and a trend towards an association of patients treated with remdesivir with a lower likelihood for in-hospital death was observed.

Our findings demonstrated that remdesivir not only is safe from the renal standpoint but might even be nephroprotective. No safety concerns were raised in patients with CKD that were treated with remdesivir either. COVID-19 initially thought to be primarily a respiratory disease, is actually a multisystem disease with several organs, often being involved including the kidneys [27,28,29]. While factors such as hemodynamic instability, shock, or hypovolemia leading to tubular injury are common mechanisms that might play a role in COVID-19-associated AKI, direct injury of the renal parenchyma by SARS-CoV-2 is likely [30,31]. Reports from autopsies of patients with COVID-19 with kidney injury revealed the presence of viral particles within both the tubular epithelium and the podocytes on electron microscopy [30]. A recent animal study showed that remdesivir may be nephroprotective in COVID-19 via effective inhibition of inflammatory immune responses, which specifically repress NLRP3 inflammasome activation in lipopolysaccharide (LPS)-activated macrophages in mice models [32]. Our findings line up with the SIMPLE-Moderate study that showed lower rates of AKI in patients receiving remdesivir compared to standard care (7% vs 10%) [4,33]. Therefore, it is likely that remdesivir improves renal outcomes both via direct inhibition of viral replication in the kidneys and through halting the inflammatory response and overall progression of COVID-19.

Our findings do not raise concerns regarding hepatotoxicity of remdesivir. Instead, the remdesivir group had lower rates of ALI compared to the non-remdesivir group but the statistical significance threshold was not reached. Our results are consistent with the ACTT-1 trial which had shown no difference in liver function test changes between remdesivir and non-remdesivir groups [4]. Similarly, a randomized controlled trial by Wang et al. reported a higher incidence of AST elevation in the placebo group compared to the remdesivir group (12% vs. 5%) [17]. Since liver injury in COVID-19 is likely caused by direct viral toxicity due to high ACE2 expression on hepatocytes and cholangiocytes [19,34], it is plausible the possibly lower ALI rate in patients who received remdesivir can be partially explained by inhibition of viral replication systemically and in the liver per se.

In the matched cohort of our study, patients that received remdesivir had a modestly lower in-hospital death rate without reaching statistical significance. The logistic regression analysis in the overall cohort revealed that remdesivir was on the verge of statistical significance to be associated with a lower likelihood for death after adjusting for important covariates including COVID-19 severity on presentation. Likely, our sample size did not provide adequate power to reveal a clear association. For instance, the RECOVERY trial which showed that Dexamethasone decreased mortality in patients with COVID-19 employed an almost seven times larger patient population [35]. The signal of possible mortality benefit depicted in our study is consistent with the results of a meta-analysis of randomized trials which demonstrated that remdesivir offered a modest decrease in mortality in patients that were on supplemental oxygen but not on mechanical ventilation [7].

Our study has several strengths. First, our patient population is of low socioeconomic status which is often underrepresented in literature. Second, we employed robust statistical analysis using the propensity-matched scoring system before estimating the treatment effects. We should acknowledge that our study has several limitations. This was a retrospective cohort involving electronic medical records, hence, there are risks related to observational bias and unmeasured confounding that cannot be mitigated by a propensity-matched scoring system [36]. However, we employed a robust independent review process and strict methodology in our efforts to minimize bias. Second, our sample size, particularly after matching, was relatively low limiting its power to detect significant associations. Third, given the relatively low sample size, we were not able to take into consideration other important variables such as concomitant treatments.

## 5. Conclusions

In conclusion, our propensity score-matched study revealed that remdesivir was safe in our patient population including patients with and without CKD and chronic liver disease. Actually, some of our findings revealed that remdesivir might be nephroprotective. In addition, a signal was noted suggesting that remdesivir might have offered a survival benefit. Overall, our real-world study findings encourage the liberal use of remdesivir in the treatment of hospitalized patients with moderate-to-severe COVID-19.

## Figures and Tables

**Table 1:** Baseline Patient Characteristics.

Characteristics	Before Matching					After Matching without Replacement				
		Remdesivir					Remdesivir			
	Total	No	Yes			Total	No	Yes		
	*n* = 927	*n* = 500	*n* = 427	*p*-Value	SMD	*n* = 248	*n* = 124	*n* = 124	*p*-Value	SMD
Gender—*n* (%)				0.285	0.070				0.525	0.080
Male	447 (48.2)	233 (46.6)	214 (50.1)			127 (51.2)	61 (49.2)	66 (53.2)		
Female	480 (51.8)	267 (53.4)	213 (49.9)			121 (48.8)	63 (50.8)	58 (46.8)		
Age—median (IQR)	61.0 (47.0–73.0)	59.0 (39.5–73.0)	63.0 (53.0–73.0)	<0.001	0.304	62.00 (50.5–73.5)	64.50 (51.0–74.0)	62.00 (49.0–72.5)	0.569	0.072
Age Category—*n* (%)				<0.001	0.249				0.801	0.075
18–44	195 (21.0)	148 (29.6)	47 (11.0)			40 (16.1)	19 (15.3)	21 (16.9)		
45–54	129 (13.9)	55 (11.0)	74 (17.3)			42 (16.9)	21 (16.9)	21 (16.9)		
55–64	198 (21.4)	93 (18.6)	105 (24.6)			50 (20.2)	22 (17.7)	28 (22.6)		
65–74	203 (21.9)	91 (18.2)	112 (26.2)			61 (24.6)	34 (27.4)	27 (21.8)		
≥75	202 (21.8)	113 (22.6)	89 (20.8)			55 (22.2)	28 (22.6)	27 (21.8)		
BMI—median (IQR)	28.51 (24.4–33.5)	27.46 (23.8–31.9)	30.02 (25.5–34.5)	<0.001	0.365	28.19 (24.4–33.7)	28.09 (25.5–33.5)	28.75 (23.5–33.8)	0.871	0.021
BMI Category—*n* (%)				<0.001	0.349				0.022	0.090
<25	259 (28.3)	165 (33.7)	94 (22.1)			64 (25.8)	25 (20.2)	39 (31.5)		
25–29.9	273 (29.8)	158 (32.2)	115 (27.0)			83 (33.5)	51 (41.1)	32 (25.8)		
≥30	384 (41.9)	167 (34.1)	217 (50.9)			101 (40.7)	48 (38.7)	53 (42.7)		
HTN—*n* (%)				<0.001	0.267				0.609	0.064
No	400 (43.2)	246 (49.2)	154 (36.1)			110 (44.4)	53 (42.7)	57 (46.0)		
Yes	527 (56.9)	254 (50.8)	273 (63.9)			138 (55.7)	71 (57.3)	67 (54.0)		
HLD—*n* (%)				0.582	0.036				0.780	0.035
No	681 (73.5)	371 (74.2)	310 (72.6)			176 (80.0)	89 (71.8)	87 (70.2)		
Yes	246 (26.5)	129 (25.8)	117 (27.4)			72 (29.0)	35 (28.2)	37 (29.8)		
DM—*n* (%)				0.004	0.188				0.372	0.113
No	567 (61.2)	327 (65.4)	240 (56.2)			135 (54.4)	64 (51.6)	71 (57.3)		
Yes	360 (38.8)	173 (34.6)	187 (43.8)			113 (45.6)	60 (48.4)	53 (42.7)		
CAD—*n* (%)				0.926	0.006				0.718	0.045
No	834 (90.1)	449 (90.0)	385 (90.2)			212 (85.5)	107 (86.3)	105 (84.7)		
Yes	92 (9.9)	50 (10.0)	42 (9.8)			36 (14.5)	17 (13.7)	19 (15.3)		
CHF—*n* (%)				0.518	0.042				1.000	0.000
No	823 (88.8)	447 (89.4)	376 (88.1)			214 (86.3)	107 (86.3)	107 (86.3)		
Yes	104 (11.2)	53 (10.6)	51 (11.9)			34 (13.7)	17 (13.7)	17 (13.7)		
Stroke—*n* (%)				0.052	0.128				0.527	0.080
No	846 (91.3)	448 (89.6)	398 (93.2)			223 (89.9)	113 (91.1)	110 (88.7)		
Yes	81 (8.7)	52 (10.4)	29 (6.8)			25 (10.1)	11 (8.9)	14 (11.3)		
CKD—*n* (%)				0.006	0.133				0.040	0.088
No	809 (87.5)	433 (87.0)	376 (88.1)			199 (80.2)	103 (83.1)	96 (77.4)		
IIIA	53 (5.7)	20 (4.0)	33 (7.7)			22 (8.9)	5 (4.0)	17 (13.7)		
IIIB	25 (2.7)	16 (3.2)	9 (2.1)			10 (4.0)	4 (3.2)	6 (4.8)		
IV	11 (1.2)	9 (1.8)	2 (0.5)			4 (1.6)	2 (1.6)	2 (1.6)		
V	18 (2.0)	15 (3.0)	3 (0.7)			11 (4.4)	9 (7.3)	2 (1.6)		
ESRD with HD—*n* (%)	9 (1.0)	5 (1.0)	4 (0.9)			2 (0.8)	1 (0.8)	1 (0.8)		
Chronic liver disease—*n* (%)				0.113	0.110				1.000	0.000
No	897 (97.0)	477 (95.8)	420 (98.4)			240 (96.8)	120 (96.8)	120 (96.8)		
Alcoholic hepatitis	25 (2.7)	19 (3.8)	6 (1.4)			8 (3.2)	4 (3.2)	4 (3.2)		
HepB	1 (0.1)	1 (0.2)	0 (0.0)							
HepC	2 (0.2)	1 (0.2)	1 (0.2)							
Cirrhosis—*n* (%)				0.123	0.129				0.845	0.038
No	911 (98.4)	487 (97.6)	424 (99.3)			243 (98.0)	121 (97.6)	122 (98.4)		
Compensated	10 (1.1)	8 (1.6)	2 (0.5)			3 (1.2)	2 (1.6)	1 (0.8)		
Decompensated	5 (0.5)	4 (0.8)	1 (0.2)			2 (0.8)	1 (0.8)	1 (0.8)		
Chronic alcohol use disorder—*n* (%)				0.002	0.236				0.485	0.021
No	882 (95.4)	464 (93.2)	418 (97.9)			234 (94.4)	118 (95.2)	116 (93.6)		
In remission	22 (2.4)	16 (3.2)	6 (1.4)			7 (2.8)	2 (1.6)	5 (4.0)		
Active	21 (2.3)	18 (3.6)	3 (0.7)			7 (2.8)	4 (3.2)	3 (2.4)		
COVID-19 severity on admission—*n* (%)				<0.001	1.655				0.456	0.057
Moderate	486 (52.5)	428 (85.8)	58 (13.6)			117 (47.2)	59 (47.6)	58 (46.8)		
Severe	331 (35.8)	50 (10.0)	281 (65.8)			97 (39.1)	45 (36.3)	52 (41.9)		
Critical	109 (11.8)	21 (4.2)	88 (20.6)			34 (13.7)	20 (16.1)	14 (11.3)		

BMI in kg/m^2^. Abbreviations and symbols: BMI = body mass index; kg = kilograms; m= meter; *n* = number; IQR = interquartile range; HTN = hypertension; HLD = hyperlipidemia; DM = Diabetes Mellitus; CAD = coronary artery disease; CHF = Congestive Heart Failure; CKD: chronic kidney disease; Hep B = Hepatitis B; Hep C = Hepatitis C.

**Table 2:** Laboratory tests on presentation.

Laboratory Tests	Before Matching					After Matching without Replacement				
		Remdesivir					Remdesivir			
	Total—*n* (%)	No—*n* (%)	Yes—*n* (%)			Total—*n* (%)	No—*n* (%)	Yes—*n* (%)		
	*n* = 927	*n* = 500	*n* = 427	*p*-Value	SMD	*n* = 248	*n* = 124	*n* = 124	*p*-Value	SMD
BUN (mg/dL)—median (IQR)	15.00 (11.0–24.0)	14.00 (10.0–22.0)	15.00 (11.0–25.0)	0.081	0.115	16.00 (11.0–29.0)	16.00 (11.0–33.0)	15.00 (11.0–27.0)	0.200	0.164
Cr (mg/dL)—median (IQR)	1.00 (0.8–1.3)	0.90 (0.7–1.3)	1.00 (0.8–1.3)	0.782	0.018	1.10 (0.8–1.6)	1.10 (0.8–1.6)	1.00 (0.8–1.5)	0.157	0.181
Albumin (g/dL)—median (IQR)	3.80 (3.5–4.2)	3.90 (3.5–4.3)	3.80 (3.5–4.0)	0.005	0.189	3.80 (3.3–4.1)	3.70 (3.2–4.1)	3.80 (3.5–4.1)	0.559	0.075
Total Bilirubin (mg/dL)—median (IQR)	0.40 (0.3–0.6)	0.40 (0.3–0.7)	0.40 (0.3–0.5)	0.002	0.214	0.40 (0.3–0.6)	0.40 (0.3–0.7)	0.30 (0.2–0.5)	0.023	0.297
ALP (U/L)—median (IQR)	79.00 (62.0–109.0)	85.00 (64.0–119.0)	75.00 (58.0–97.0)	0.002	0.214	79.00 (60.0–111.0)	82.00 (61.0–133.0)	74.00 (58.0–93.0)	0.007	0.359
AST (U/L)—median (IQR)	34.00 (24.0–60.0)	29.00 (21.0–49.0)	42.00 (28.0–64.0)	0.251	0.079	38.00 (27.0–63.0)	34.00 (25.0–62.0)	42.00 (30.0–63.0)	0.029	0.281
ALT (U/L)—median (IQR)	26.00 (16.0–45.5)	23.50 (14.0–42.0)	28.00 (19.0–48.0)	0.256	0.078	29.00 (18.0–53.0)	29.50 (17.0–67.0)	27.00 (20.0–46.0)	0.019	0.303

Results of liver and kidney function tests in patients who received remdesivir and patients who did not receive remdesivir, before and after matching are provided. Abbreviations and symbols: *n* = number; mg = milligram; dL = deciliter; U/L = Unit/Litre BUN = Blood urea nitrogen; Cr = Creatinine; ALP = Alkaline phosphatase; AST = Aspartate aminotransferase; ALT = Alanine aminotransferase; IQR = Interquartile range.

**Table 3:** In-hospital outcomes.

Outcomes	Before Matching					After Matching without Replacement				
		Remdesivir					Remdesivir			
	Total—*n* (%)	No—*n* (%)	Yes—*n* (%)			Total—*n* (%)	No—*n* (%)	Yes– *n* (%)		
	*n* = 927	*n* = 500	*n* = 427	*p*-Value	SMD	*n* = 248	*n* = 124	*n* = 124	*p*-Value	SMD
Intubation—*n* (%)				<0.001	0.373				0.078	0.22
No	809 (87.3)	465 (93.0)	344 (80.6)			210 (84.7)	100 (80.7)	110 (88.7)		
Yes	118 (12.7)	35 (7.0)	83 (19.4)			38 (15.3)	24 (19.4)	14 (11.3)		
Admission to ICU—*n* (%)				<0.001	0.451				0.548	0.08
No	748 (80.7)	444 (88.8)	304 (71.2)			190 (76.6)	93 (75.0)	97 (78.2)		
Yes	179 (19.3)	56 (11.2)	123 (28.8)			58 (23.4)	31 (25.0)	27 (21.8)		
Death—*n* (%)				<0.001	0.352				0.593	0.07
No	817 (88.1)	467 (93.4)	350 (82.0)			211 (85.1)	104 (83.9)	107 (86.3)		
Yes	110 (11.9)	33 (6.6)	77 (18.0)			37 (14.9)	20 (16.1)	17 (13.7)		
AKI during hospitalization				0.056	0.125				0.042	0.26
No	805 (86.8)	444 (88.8)	361 (84.5)			206 (83.1)	97 (78.2)	109 (87.9)		
Yes	122 (13.2)	56 (11.2)	66 (15.5)			42 (16.9)	27 (21.8)	15 (12.1)		
New dialysis during hospitalization				0.055	0.123				0.156	0.180
No	918 (99.0)	498 (99.6)	420 (98.4)			246 (99.2)	122 (98.4)	124 (100.0)		
Yes	9 (1.0)	2 (0.4)	7 (1.6)			2 (0.8)	2 (1.6)	0 (0.0)		
ALI during hospitalization				0.150	0.094				0.067	0.23
No	851 (91.8)	465 (93.0)	386 (90.4)			221 (89.1)	106 (85.5)	115 (92.7)		
Yes	76 (8.2)	35 (7.0)	41 (9.6)			27 (10.9)	18 (14.5)	9 (7.3)		

(1) The outcomes are presented as *n* (%), (2) Presence or absence of each outcome is indicated by ‘yes’ and ‘no’ below it, (3) Before Matching, out of a total of 927 patients, 427 received remdesivir and 500 did not. After matching, a total of 248 patients were divided into two equal groups based on the administration of remdesivir. Abbreviations and symbols: *n* = number; ICU = Intensive Care Unit; AKI = Acute Kidney Injury; ALI = Acute Liver Injury.

**Table 4:** Subgroup Analysis for patients with chronic kidney disease and chronic liver disease.

	Before Matching					After Matching without Replacement				
		Remdesivir					Remdesivir			
	Total—*n* (%)	No—*n* (%)	Yes—*n* (%)			Total—*n* (%)	No—*n* (%)	Yes—*n* (%)		
**Patients with CKD**	*n* = 107	*n* = 60	*n* = 47	*p*-value	SMD	*n* = 37	*n* = 15	*n* = 22	*p*-value	SMD
AKI during hospitalization				0.141	0.291				0.967	0.013
No	84 (78.5)	44 (73.3)	40 (85.1)			27 (73.0)	11 (73.3)	16 (72.4)		
Yes	23 (21.5)	16 (26.7)	7 (14.9)			10 (27.0)	4 (26.7)	6 (27.3)		
**Patients with Chronic Liver Disease**	*n* = 28	*n* = 21	*n* = 7			*n* = 8	*n* = 4	*n* = 4		
ALI during hospitalization				0.111	0.872				0.285	0.707
No	22 (78.6)	15 (71.4)	7 (100.0)			7 (87.5)	3 (75.0)	4 (100.0)		
Yes	6 (21.4)	6 (28.6)	0 (0.0)			1 (12.5)	1 (25.0)	0 (0.0)		

Subgroup Analysis of patients with chronic kidney disease and chronic liver disease with and without acute kidney injury and acute liver injury respectively. Abbreviations and symbols: CKD: Chronic Kidney Disease, AKI: Acute Kidney Injury; ALI: Acute Liver Injury.

**Table 5:** Logistic Regression Analysis for Acute Kidney Injury.

Outcomes	Before Matching		After Matching without Replacement	
	Univariate Analysis	Multivariate Analysis	Univariate Analysis	Multivariate Analysis
		*n* = 926		*n* = 248
	OR, 95% CI, *p*-Value	OR, 95% CI, *p*-Value	OR, 95% CI, *p*-Value	OR, 95% CI, *p*-Value
Female	0.73 (0.50–1.08) *p* = 0.113		0.84 (0.43–1.64) *p* = 0.614	
Age Category	1.57 (1.36–1.80) *p* < 0.001	1.36 (1.15–1.62) *p* < 0.001	1.42 (1.09–1.85) *p* = 0.009	1.26 (0.93–1.69) *p* = 0.131
BMI	1.02 (0.99–1.04) *p* = 0.134		1.02 (0.98–1.06) *p* = 0.290	
Hypertension	2.88 (1.85–4.50) *p* < 0.001	1.82 (1.08–3.07) *p* = 0.026	2.27 (1.10–4.68) *p* = 0.027	1.67 (0.79–3.50) *p* = 0.177
Hyperlipidemia	1.48 (0.99–2.23) *p* = 0.059		1.45 (0.72–2.93) *p* = 0.298	
Diabetes	1.97 (1.34–2.89) *p* = 0.001	1.09 (0.69–1.74) *p* = 0.712	1.75 (0.90–3.43) *p* = 0.102	
CAD	1.09 (0.59–2.04) *p* = 0.775		1.50 (0.63–3.57) *p* = 0.364	
CHF	2.52 (1.54–4.13) *p* < 0.001	1.44 (0.84–2.46) *p* = 0.181	2.82 (1.25–6.38) *p* = 0.013	1.59 (0.67–3.78) *p* = 0.297
Stroke	1.29 (0.69–2.42) *p* = 0.422		1.26 (0.44–3.57) *p* = 0.668	
CKD or ESRD	1.17 (1.00–1.37) *p* = 0.051		1.57 (0.72–3.40) *p* = 0.254	
Chronic liver disease	1.03 (0.60–1.78) *p* = 0.906		3.09 (0.71–13.51) *p* = 0.134	
Cirrhosis	1.40 (0.63–3.08) *p* = 0.410		2.32 (0.57–9.46) *p* = 0.241	
COVID-19 severity on admission	2.86 (2.14–3.81) *p* < 0.001	3.69 (2.61–5.21) *p* < 0.001	3.28 (1.94–5.53) *p* < 0.001	2.99 (1.76–5.09) *p* < 0.001
Remdesivir	1.45 (0.99–2.12) *p* = 0.057	0.40 (0.24–0.67) *p* = 0.000	0.49 (0.25–0.98) *p* = 0.045	0.48 (0.23–1.01) *p* = 0.054

(1) BMI in kg/m^2^, (2) age in years. Abbreviations and symbols: BMI: Body Mass Index, CAD: Coronary Artery Disease, CHF: Congestive Heart Failure, CKD: Chronic Kidney Disease; ESRD: End-Stage Renal Disease, OR: Odd’s ratio; CI: Confidence index.

**Table 6:** Logistic Regression Analysis for Acute Liver Injury.

Outcomes	Before Matching		After Matching without Replacement	
	Univariate Analysis	Multivariate Analysis	Univariate Analysis	Multivariate Analysis
		*n* = 924		*n* = 248
	OR, 95% CI, *p*-Value	OR, 95% CI, *p*-Value	OR, 95% CI, *p*-Value	OR, 95% CI, *p*-Value
Female sex	0.45 (0.28–0.74) *p* = 0.002	0.58 (0.35–0.98) *p* = 0.041	0.69 (0.31–1.56) *p* = 0.378	
Age Category	0.90 (0.78–1.04) *p* = 0.151		0.84 (0.64–1.09) *p* = 0.191	
BMI	1.01 (0.98–1.04) *p* = 0.446		1.00 (0.96–1.04) *p* = 0.963	
Hypertension	0.52 (0.33–0.84) *p* = 0.008	0.57 (0.32–1.02) *p* = 0.060	0.60 (0.27–1.35) *p* = 0.219	
Hyperlipidemia	0.44 (0.23–0.86) *p* = 0.015	0.66 (0.32–1.35) *p* = 0.255	0.84 (0.34–2.09) *p* = 0.707	
Diabetes	0.58 (0.34–0.97) *p* = 0.039	0.67 (0.36–1.23) *p* = 0.199	0.56 (0.24–1.31) *p* = 0.182	
CAD	0.48 (0.17–1.35) *p* = 0.164		0.71 (0.20–2.51) *p* = 0.597	
CHF	0.66 (0.28–1.56) *p* = 0.341		0.47 (0.11–2.10) *p* = 0.324	
Stroke	1.07 (0.47–2.40) *p* = 0.879		1.66 (0.52–5.26) *p* = 0.392	
CKD or ESRD	0.69 (0.31–1.54) *p* = 0.363		0.91 (0.33–2.56) *p* = 0.864	
Chronic liver disease	1.49 (0.89–2.51) *p* = 0.131		1.18 (0.14–9.98) *p* = 0.882	
Cirrhosis	0.69 (0.17–2.88) *p* = 0.613		cannot be estimated	
COVID-19 severity on admission	2.19 (1.57–3.05) *p* < 0.001	2.75 (1.81–4.16) *p* < 0.001	2.20 (1.23–3.92) *p* = 0.008	2.16 (1.22–3.82) *p* = 0.008
Remdesivir	1.41 (0.88–2.26) *p* = 0.152	0.68 (0.35–1.30) *p* = 0.241	0.46 (0.20–1.07) *p* = 0.072	0.47 (0.20–1.11) *p* = 0.087

(1) BMI in kg/m^2^, (2) age in years. Abbreviations and symbols: BMI: Body Mass Index, CAD: Coronary Artery Disease, CHF: Congestive Heart Failure, CKD: Chronic Kidney Disease; ESRD: End-Stage Renal Disease, OR: Odd’s ratio; CI: Confidence index.

**Table 7:** Logistic Regression Analysis for In-hospital Mortality.

Outcomes	Before Matching		After Matching without Replacement	
	Univariate Analysis	Multivariate Analysis	Univariate Analysis	Multivariate Analysis
		*n* = 924		*n* = 248
	OR, 95% CI, *p*-Value	OR, 95% CI, *p*-Value	OR, 95% CI, *p*-Value	OR, 95% CI, *p*-Value
Female sex	0.72 (0.48–1.07) *p* = 0.107		0.87 (0.43–1.76) *p* = 0.708	
Age Category	1.49 (1.28–1.72) *p* < 0.001	1.36 (1.11–1.67) *p* = 0.003	1.22 (0.94–1.59) *p* = 0.130	
BMI	1.01 (0.99–1.04) *p* = 0.295		1.02 (0.97–1.06) *p* = 0.484	
Hypertension	1.32 (0.87–1.99) *p* = 0.186		1.20 (0.59–2.45) *p* = 0.614	
Hyperlipidemia	1.04 (0.67–1.63) *p* = 0.852		1.40 (0.67–2.93) *p* = 0.378	
Diabetes	1.20 (0.80–1.80) *p* = 0.373		1.31 (0.65–2.65) *p* = 0.445	
CAD	0.90 (0.45–1.78) *p* = 0.753		0.91 (0.33–2.51) *p* = 0.851	
CHF	2.09 (1.23–3.54) *p* = 0.006	1.17 (0.62–2.22) *p* = 0.627	1.96 (0.81–4.76) *p* = 0.136	
Stroke	1.80 (0.99–3.27) *p* = 0.056		4.83 (1.97–11.87) *p* = 0.001	3.34 (0.94–11.80) *p* = 0.061
CKD or ESRD	1.29 (1.10–1.51) *p* = 0.001	1.29 (1.08–1.54) *p* = 0.006	1.25 (0.98–1.60) *p* = 0.071	
Chronic liver disease	0.96 (0.52–1.76) *p* = 0.889		3.64 (0.83–15.96) *p* = 0.087	
Cirrhosis	0.88 (0.32–2.42) *p* = 0.799		1.69 (0.48–5.95) *p* = 0.410	
COVID-19 severity on admission	8.23 (5.46–12.43) *p* < 0.001	9.25 (5.99–14.29) *p* < 0.001	10.89 (4.50–26.36) *p* < 0.001	10.24 (4.14–25.31) *p* < 0.001
Remdesivir	3.11 (2.02–4.79) *p* < 0.001	0.57 (0.32–1.01) *p* = 0.053	0.83 (0.41–1.67) *p* = 0.594	0.97 (0.42–2.22) *p* = 0.941

(1) BMI in kg/m^2^, (2) age in years. Abbreviations and symbols: BMI: Body Mass Index, CAD: Coronary Artery Disease, CHF: Congestive Heart Failure, CKD: Chronic Kidney Disease; ESRD: End-Stage Renal Disease, OR: Odd’s ratio; CI: Confidence index.

## Data Availability

Not applicable.

## Supplementary Materials

The following supporting information can be downloaded at: https://www.mdpi.com/article/10.3390/jcm11113132/s1, Table S1: Acute Kidney Injury per Stage.
