# Supplementary material for: Remdesivir in the Treatment of COVID-19: A Propensity Score-Matched Analysis from a Public Hospital in New York City Assessing Renal and Hepatic Safety
Source: J Clin Med. 2022 May 31;11(11):3132. doi: 10.3390/jcm11113132 (PMC9181125; doi:10.3390/jcm11113132)
Supplement: Supplementary file 1 [file jcm-11-03132-s001.zip › jcm-1672339-supplementary.pdf]

Supplementary Table S1: Acute Kidney Injury per Stage

	Before matching					After Matching without Replacement				
		Remdesivir					Remdesivir			
	Total	No	Yes			Total	No	Yes		
	N=122	N=56	N=66	p-value	SMD	N=42	N=27	N=15	p-value	SMD
AKI Stage	n (%)	n (%)	n (%)	0.037	0.251	n (%)	n (%)	n (%)	0.905	0.049
1	73 (59.84)	39 (69.64)	34 (51.52)			29 (69.05)	19 (70.37)	10 (66.67)		
2	16 (13.11)	3 (5.36)	13 (19.70)			2 (4.76)	1 (3.70)	1 (6.67)		
3	33 (27.05)	14 (25.00)	19 (28.79)			11 (26.11)	7 (25.93)	4 (26.67)		

1) Presence or absence of each outcome is indicated by 'yes' and 'no' below it, 3) AKI staging was performed per KDIGO guidelines. Abbreviations and symbols: N, n = number; AKI = Acute Kidney Injury
